# Genetically modified ZIKA virus as a microRNA-sensitive oncolytic virus against central nervous system tumors

**DOI:** 10.1016/j.ymthe.2024.01.006

**Published:** 2024-01-11

**Authors:** Gabriela Machado Novaes, Caroline Lima, Carla Longo, Pedro Henrique Machado, Thais Peron Silva, Giovanna Gonçalves de Oliveira Olberg, Diego Grando Módolo, Márcia Cristina Leite Pereira, Tiago Goss Santos, Mayana Zatz, David Lagares, Marcelo de Franco, Paulo Lee Ho, Harry Bulstrode, Oswaldo Keith Okamoto, Carolini Kaid

**Affiliations:** 1 Vyro Bio Inc., Sao Paulo 05508-000, Brazil; 2 International Research Center/CIPE, A.C. Camargo Cancer Center, Sao Paulo 01508-010, Brazil; 3 Human Genome and Stem Cell Research Center, Department of Genetics and Evolutionary Biology, Institute of Biosciences, University of Sao Paulo, Sao Paulo 05508-900, Brazil; 4 Center for Immunology and Inflammatory Diseases, Division of Rheumatology, Allergy and Immunology, Massachusetts General Hospital, Harvard Medical School, Boston, MA 02114, USA; 5 Pasteur Institute, Diagnostic Section, Sao Paulo 01311-000, Brazil; 6 Butantan Institute, BioIndustrial Center, Sao Paulo 05503-900, Brazil; 7 Wellcome-Medical Research Council Cambridge Stem Cell Institute, Cambridge Biomedical Campus, University of Cambridge, Cambridge CB2 0AW, UK

**Keywords:** central nervous system tumors, miRNA-sensitive oncolytic virus, virus genetic engineering, Zika virus, cancer stem cell, glioblastoma, medulloblastoma, AT/RT, oncolytic therapy, immunotherapy

## Abstract

Here we introduce a first-in-class microRNA-sensitive oncolytic Zika virus (ZIKV) for virotherapy application against central nervous system (CNS) tumors. The described methodology produced two synthetic modified ZIKV strains that are safe in normal cells, including neural stem cells, while preserving brain tropism and oncolytic effects in tumor cells. The microRNA-sensitive ZIKV introduces genetic modifications in two different virus sites: first, in the established 3′UTR region, and secondly, in the ZIKV protein coding sequence, demonstrating for the first time that the miRNA inhibition systems can be functional outside the UTR RNA sites. The total tumor remission in mice bearing human CNS tumors, including metastatic tumor growth, after intraventricular and systemic modified ZIKV administration, confirms the promise of this virotherapy as a novel agent against brain tumors—highly deadly diseases in urgent need of effective advanced therapies.

## Graphical abstract

**Figure:**
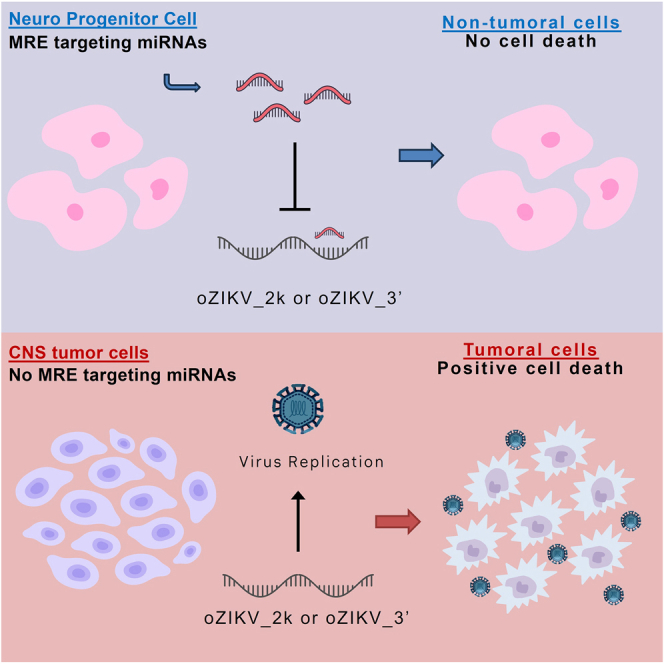

Kaid and colleagues developed the first-in-class microRNA-sensitive oncolytic Zika virus, demonstrating that the inhibition systems can be functional outside the UTR RNA sites, for virotherapy application against CNS tumors. The methodology described produced modified ZIKV strains that are safe in normal cells, while preserving brain tropism and oncolytic effects.

## Introduction

Oncolytic virotherapy is a promising therapeutic class that employs active viruses to replicate and selectively destroy the cancer cells.^1^ Malignant CNS tumors are one of the deadliest tumors and available therapies demonstrate limited efficacy.^2^ Until now, three oncolytic virotherapies have recently received marketing authorization for melanoma, head/neck cancer, and malignant glioma as target indications.^3^^,^^4^^,^^5^ These approved drugs involve genetically modified viruses, Herpes simplex virus 1 (HSV-1) and Adenovirus Serotype 5 (Ad5), with removal and insertion of sequences to improve virus cancer cell specificity, oncolytic potency, delivery, and virus spread.^6^ However, developing a modified virus with all these optimized features with fine adjustment of tumor oncolysis and host immune response has been a challenge, especially because of the safety concerns of using replication-competent viruses.^7^

Recent studies showed that the Zika virus (ZIKV) exhibits an oncolytic effect against CNS tumors.^8^^,^^9^^,^^10^ The wild-type ZIKV offers key attributes of effective virotherapy, including effectiveness, rapid response, direct targeting, minimal side effects, and fewer clinical interventions.^11^^,^^12^^,^^13^ Alongside these oncolytic therapy attributes, ZIKV prominently infects neural stem and progenitor cells (NPCs) and disrupts key cellular processes, leading to massive cell death and impaired self-renewal, resulting in severe CNS abnormalities in neonates, including Zika congenital syndrome with microcephaly.^14^^,^^15^ Therefore, the clinical application of ZIKV as virotherapy will depend on genetic modification to guarantee therapy safety.

Here, we increased safety and virus selectivity by generating the first modified ZIKV using miRNA-sensitive technology, which controls viral replication in normal cells through the introduction of engineered target sites, or miRNA response elements (MREs). The MREs were inserted into the canonical 3′ UTR site, but also for the first time in the ZIKV open reading frame, by the challenging approach of cloning in the bacterial artificial chromosome (BAC), a strategy designed to overcome the toxicity of the genes prM-E-NS1 in bacteria during the cloning procedure.^16^

The two modified ZIKV strains generated were proven to be safe in normal cells, with no decrease in cell viability after infection, and oncolytic in tumor cell lines including embryonal CNS tumors, glioblastoma, prostate cancer, and triple-negative breast cancer. When systemically injected in Balb/C Nude mice bearing CNS tumor, the modified ZIKV crossed the blood brain barrier (BBB) and improved the survival rate when compared not only with the control group but also with wild-type ZIKV, confirming the technology safety. The results presented here demonstrate development of a breakthrough biotechnology with the potential to be the first-in-class oncolytic ZIKV (oZIKV) virotherapy, and to offer an effective treatment strategy against lethal CNS tumors with increased safety.

## Results

### Evaluation of miRs expression in commercial NPC cells, iPS-derived NPC, tumoral and non-tumoral cells

**Figure 1:**
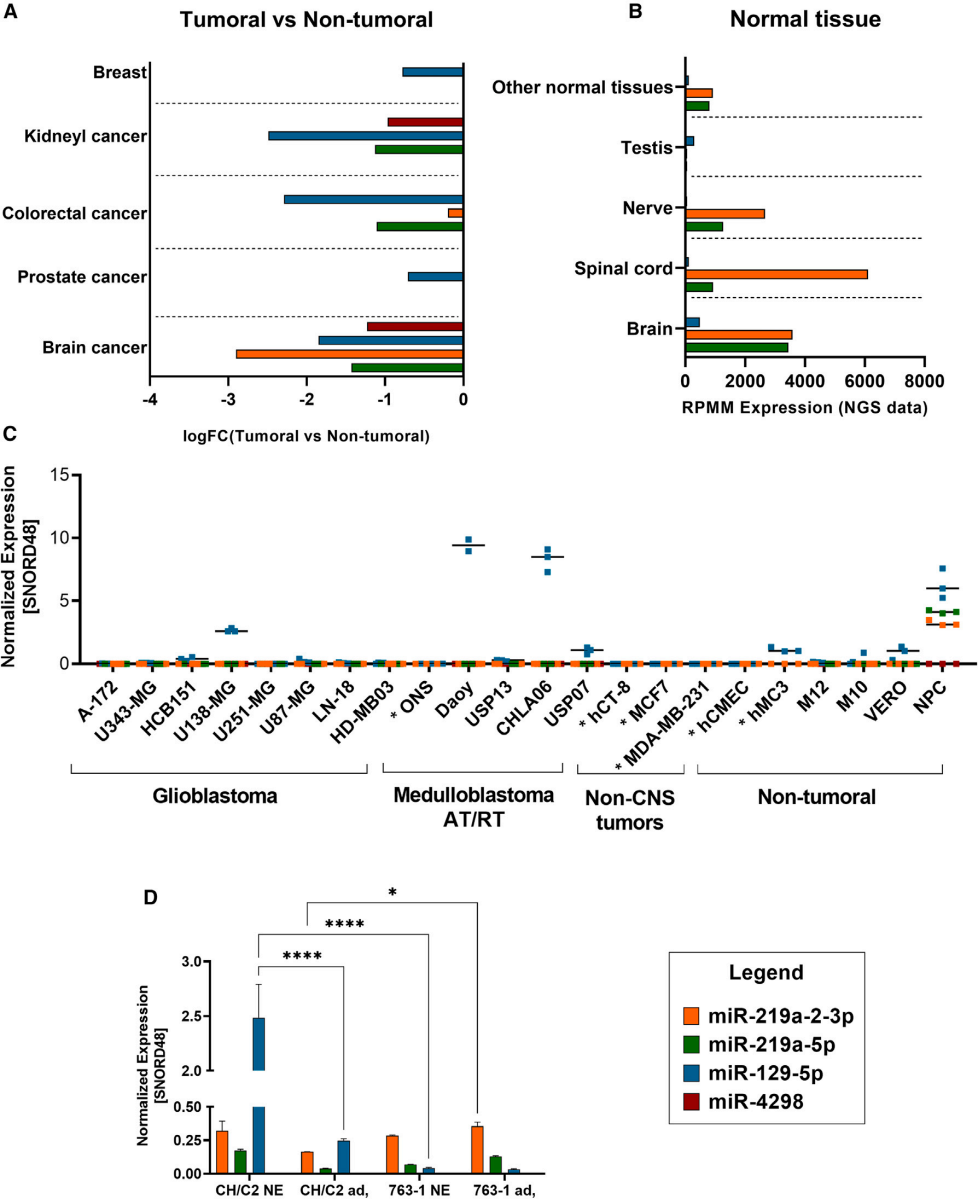
miRNAs expression profile (A) *In silico* analysis of miRs (miR-219a-2-3p, miR-219a-5p, miR129-5p, and miR4298) in tumoral vs. non-tumoral samples based on a Database of Differentially Expressed miRNAs in Human Cancers (dbDEMC). (B) miR expression in healthy tissues based on TissueAtlas- Human miRNA Patterns database. (C) RT-PCR quantification of miR expression normalized to SNORD48 in tumoral (glioblastoma, medulloblastoma, AT/RT, and non-CNS tumors) and non-tumoral (human mesenchymal, monkey kidney, microglia, cerebral microvessel, and neural progenitor cell line from lonza: NPC) cell lines. Cells indicated with ∗ were evaluated only for miRs miR-219a-2-3p and miR129-5p. Each dot represents one biological replicate, and horizontal lines indicate the mean of data in each group (n = 3). (D) RT-PCR quantification of miRs expression normalized to SNORD48 in neural progenitor cells derived from human iPS isolated from a patient with Congenital ZIKA Syndrome (763-1) and control (CH/C2) growing as neurosphere (NE) and 2D culture (ad). Each bar represents one biological replicate plotted with mean and standard deviation (n = 3). Significance determined by one-way ANOVA Tukey’s multiple-comparison test. ∗∗∗∗p < 0.0001, ∗p < 0.05.

To control ZIKV replication in non-tumor cells without losing oncolytic effect, we inserted miRNA response elements (MRE), recognized by miRNAs expressed in healthy tissues especially neural cells, but downregulated in tumors. These were cloned into the ZIKV genome to promote the degradation of these sequences in healthy tissue, but not in tumor cells for safety concerns.^17^ For that, we first searched public databases for tumor-suppressed miRNAs highly expressed in healthy tissues. Figures 1A and 1B show *in silico* analysis of four human miRNAs, miR-4298 (CUGGGACAGGAGGAGGAGGCAG), miR-129-5p (CUUUUUGCGGUCUGGGCUUGC), miR-219a-2-3p (AGAAUUGUGGCUGGACAUCUGU), and miR-219a-5p (UGAUUGUCCAAACGCAAUUCU) demonstrating the expression profile required, especially overexpression in neural healthy tissue (brain, nerve, and spinal cord) and downregulation in brain tumor (Figures 1A and 1B; Table S1).

To confirm the *in silico* data, RT-PCR expression analysis was performed on seven glioblastoma cell lines (A-172, U343-MG, HCB151, U138-MG, U251-MG, U87-MG, and LN-18), five embryonal CNS tumor cell lines (medulloblastoma: HD-MB03, Daoy, USP13; AT/RT: CHLA06 and USP07), three non-tumoral human strains (mesenchymal: M12 and M10; commercial neural progenitor-Lonza©: NPC) and the Vero cell, renal epithelial cells derived from African green monkeys, which was used to produce the modified virus. Figure 1C shows that the expression profile observed in the *in silico* study was confirmed in cells for the miR-219a-2-3p, miR-219a-5p, and miR-129-5p.

Since NPCs are a preferred cell target for ZIKV, a more detailed miRNA expression analysis was performed using NPC derived from iPS cells isolated from susceptible human patients who developed Congenital ZIKA Syndrome (ZCS), leading to microcephaly due to ZIKV infection during pregnancy,^18^ varying the cell culture conditions: 2D adherent culture and 3D neurosphere culture. Figure 1D shows that miR-219a-2-3p, miR-219a-5p, and miR-129-5p demonstrated positive expression in both controls (CH/C2) and ZCS patient (763-1) NPC cells, with miR-129-5p the target that reached the highest expression levels. The miR-4298 did not show positive expression in any condition for either cell line or in commercial NPCs (Lonza) (Figure 1D). The CH/C2 iPS-derived NPC cell line showed higher expression of miR-129-5p when cultured under spheroid condition, since the NE culture favors progenitor cell growth.^19^ The miRNA miR-129-5p and miR-219a-2-3p presented the most desirable profile for miRNA selection, specifically high expression in NPC cell lines and absent expression in most tumor cell lines.

### Generation of partial ZIKV constructs: Cloning strategy

**Figure 2:**
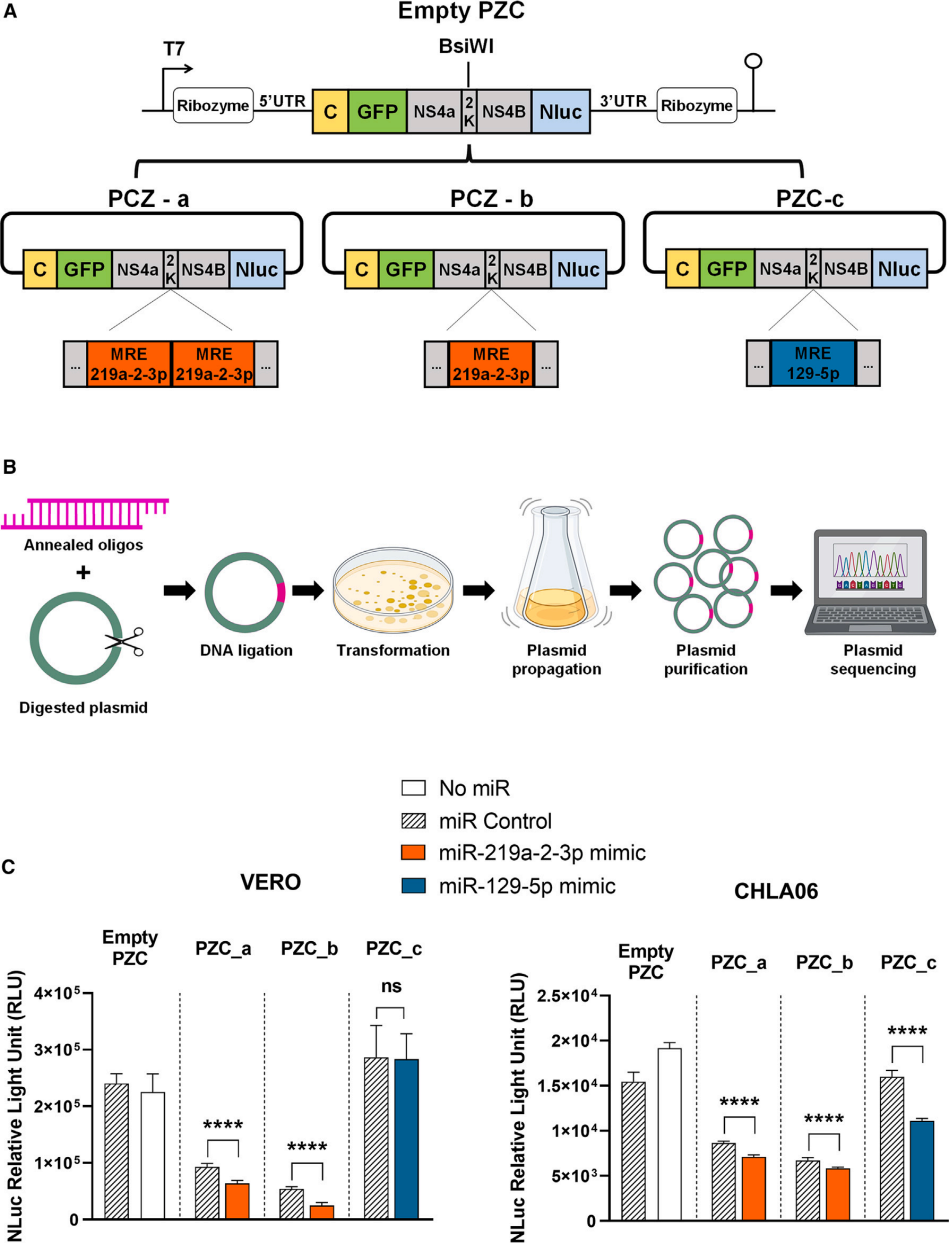
PZC design and MRE validation at the protein level (A) Representative image of a non-replicative viral vector model (PZC) construct containing two ribozymes, ZIKV partial capsid sequence (yellow), GFP (green), non-structural ZIKV partial sequence (gray), Nluc (blue), and de MRE designed for miR-129-5p (blue) and miR-219a-2-3p (orange). (B) Schematic representation of MRE oligos cloning strategy detailed in the methods section. (C) Nluc quantification in PZC containing different MREs (PZC_a: miR-219a-2-3p with two MRE copies; PZC_b: miR-219a-2-3p with one copy of MRE; PZC_c: miR-129-5p) after corresponding miRNA overexpression in Vero (left) and CHLA-06-AT/RT (right) cell line. Each bar represents one biological replicate plotted with mean and standard deviation (n = 3). Significance determined by one-way ANOVA Tukey’s multiple-comparison test. ∗∗∗∗p < 0.0001.

To determine the ideal MRE sequence to be inserted into oZIKV, we generated a non-replicative partial construct model (PZC), using parts of the ZIKV genome capable of encoding and encapsulating reporter genes, to test the miRNA inhibition mechanism in a simpler model. In this construction, a prokaryotic RNA polymerase (T7) was used as a promoter, a Hammerhead ribozyme at 5′UTR and a Hepatitis delta virus ribozyme sequence (HDVr) at 3′ UTR for enhancing the *in vitro* transcription and producing accurate UTR ends (Figures 2A and S1). We next inserted ZIKV genes previously described to play an important role in encapsulation of the virus, such as the UTRs and the capsid genes.^20^^,^^21^^,^^22^ The MRE sequence was inserted in the plasmid by annealing a pair of oligos with the sequence flanked by the restriction site and cloned into the PZC, as detailed in the methodology (Figure 2B). Due to the results obtained in Figure 1, we selected MREs for miRNA 129-5p and 219a-2-3p for insertion into the PZC. Moreover, to evaluate the inhibition mechanism in the presence of more MREs, clones with one or two copies of targets were generated (Figure 2A).

To mimic the native intracellular ZIKV state, the plasmid RNA was transfected into VERO and CHLA06 cell lines, with degradation secondary to miRNA overexpression visualized via NanoLuc reporter expression (Figure 2C). Overexpression of the corresponding miRNA in the CHLA06 cell line led to NanoLuc inhibition in PZC_a (duplicated MRE of miR-129-5p), PZC_b (one copy of MRE of miR-129-5p), and PZC_c (one copy of MRE of miR-219a-2-3p) constructs (Figure 2C, right). In Vero cells, inhibition was observed only in response to miR-219a-2-3p modulation (constructs PZC_a, and PZC_b) (Figure 2C, left). To analyze the activity of both miRNAs (miR-129-5p and miR-219a-2-3p) inserted in different ZIKV genome positions (canonical 3′ UTR site or open reading frame), the MRE sequence from PZC_b and PZC_c was chosen to be cloned into the virus.

### miRNA-sensitive synthetic ZIKV generation: oZIKV_2k and oZIKV_3′

**Figure 3:**
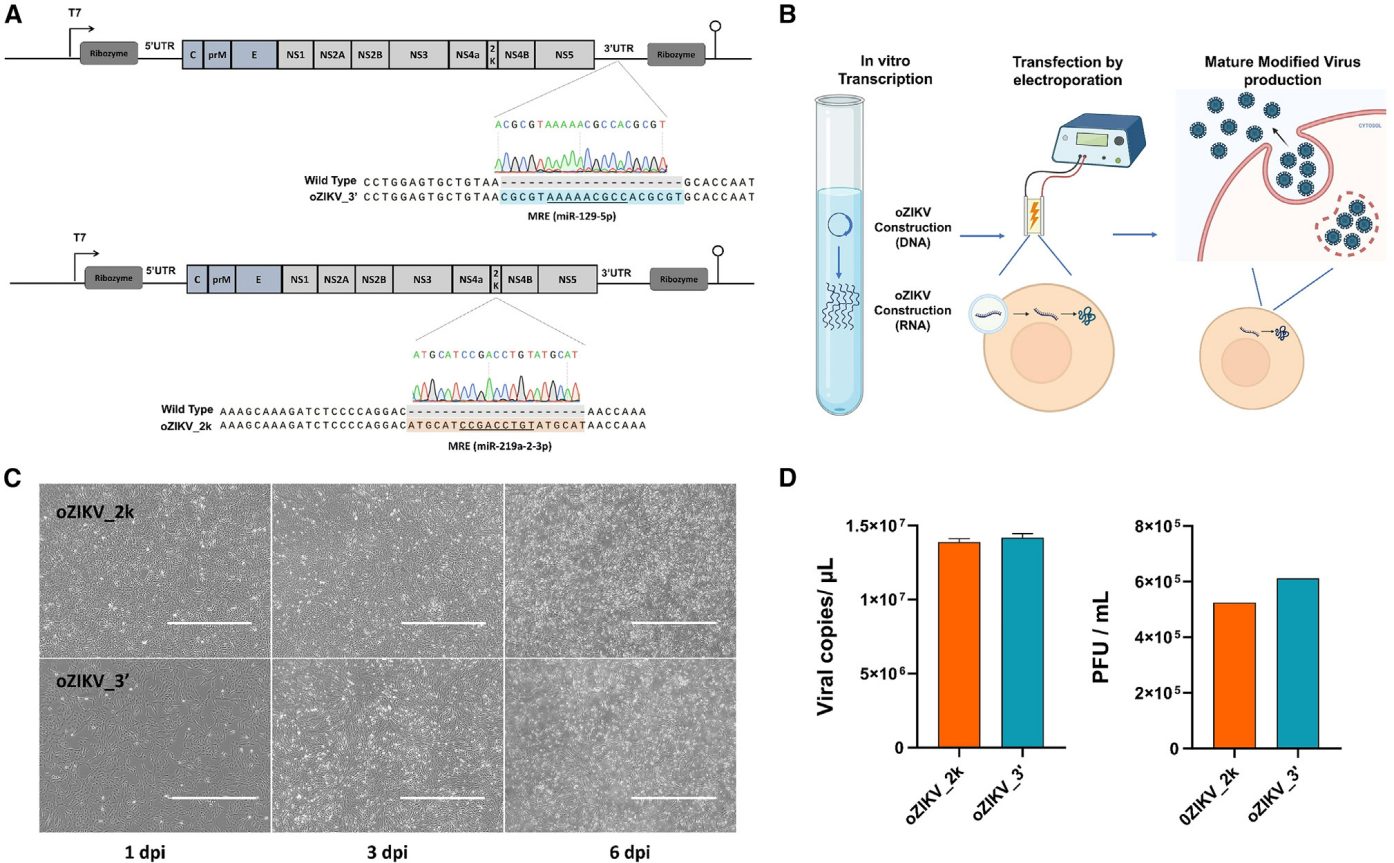
miRNA-sensitive synthetic ZIKV generation (A) Representative image of the synthetic microRNA-sensitive oZIKV constructs (oZIKV_2k and oZIKV_3′) highlighting the insertion of MRE at 3′UTR and 2k. (B) Schematic representation of synthetic microRNA-sensitive oZIKV generation process. (C) Representative images of Vero cell after oZIKV_2k and oZIKV_3′ infection at 1, 3, and 6 days post-infection (dpi). Bar scale of 1,000 μm. (D) Virus titer of culture supernatant after oZIKV_2k and oZIKV_3′ active virus production and harvest. The viral RNA copy was quantified by RT-PCR (left). Each bar represents one biological replicate plotted with mean and standard deviation (n = 3). The active virus was quantified by PFU (right).

Based on the Brazilian strain genome (NCBI ID MH882527.1) NCBI databank, we generated a synthetic miRNA-sensitive oZIKV, modified by base substitution to silence unwanted restriction sites. The ZIKV genome comprises 10,806 base pairs and was synthesized in a pCC1 low copy plasmid that maintains the stability of large genes and has controlled replication to overcome the toxicity problems of flavivirus’ cDNA in *E. coli*, carrying a T7 promoter and ribozymes positioned at the UTRs, as the partial oZIKV constructs.^23^ From this parental plasmid, we generated two different constructs: oZIKV_3′ incorporated a nine-nucleotide miR-129-5p MRE in the 3′ UTR (Figure 3A), a canonical position of MRE insertions in RNA viruses.^24^ oZIKV_2k incorporated a nine-nucleotide miR-219a-2-3p MRE in the 2k transmembrane peptide region located between NS4A and NS4B (Figure 3A).

After successful MRE insertion in the pCC1 plasmid containing the ZIKV genome by restriction cloning, confirmed by Sanger sequencing, we proceeded to *in vitro* transcription and RNA electroporation into Vero cells for mature modified virus production (Figure 3B). We observed high production of modified ZIKV 6 days after RNA transfection (Figure 3C). The synthetically modified virus produced was quantified by RT-PCR and PFU (Figure 3D). We observed a viral particle (vp) count of 1.39 × 10^7^ and 1.45 × 10^7^ copies/mL for oZIKV_2k and oZIKV_3′, respectively. The culture supernatant titer showed an infective particle (ip) amount of 5.25 × 10^5^ and 6.12 × 10^5^ PFU/mL. For production efficiency, we calculated the ratio of viral particle (vp) and infective particle (ip), resulting in a vp/ip of 26.49 and 23.66, for oZIKV_2k and oZIKV_3′, respectively. The US Food and Drug Administration regulatory agency (FDA) recommends a vp/ip ratio up to 30 for Herpes virus, whereas there are no recommendations for ZIKV, and in general it is necessary for oncolytic virus developers to determine the best vp/ip for viruses not described in the FDA guide.^25^ The vp/ip ratio obtained for oZIKV_2k and oZIKV_3′ production was lower than the limit required for the FDA, indicating an efficient production process. The synthetic and modified virus batches produced were preserved at −80°C for the next steps.

### oZIKV_2k and oZIKV_3′ modulation by miR-219a-2-3p and miR-129-5p

**Figure 4:**
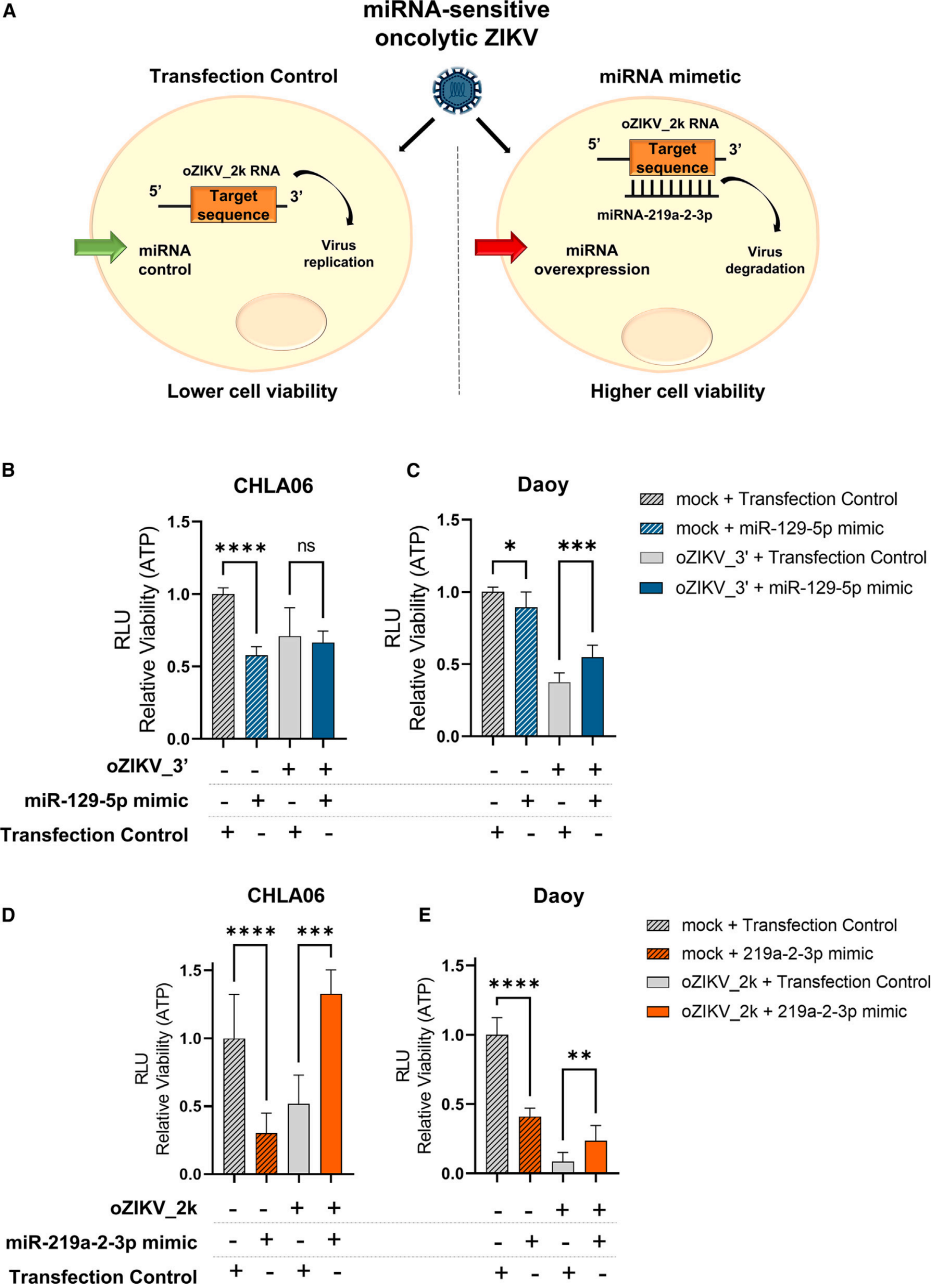
miRNA inhibition mechanism at oZIKV (A) Schematic representation of miRNA inhibition mechanism test in the modified oncolytic virus. Briefly, the corresponding miRNA was previously overexpressed in the cell line infected with miRNA-sensitive oZIKV, leading to virus RNA degradation after miRNA binding, consequently increasing cell viability. (B and C) Cell viability assay in CHLA06 (B) and Daoy (C) cell lines submitted to transient transfection with miR-129-5p or miRNA control. Twenty-four hours after transfection, the cells were infected with oZIKV_3′ at MOI 1 for CHLA06 and MOI 0.1 for Daoy. The cell viability was analyzed 5 days after infection. (D and E) Cell viability assay in CHLA06 (D) and Daoy (E) cell lines transiently transfected with miR-219a-2-3p or miRNA control. For (B), (C), (D), and (E), 24 h after transient transfection, the cells were infected with the corresponding miRNA-sensitive oZIKV (oZIKV_3′ for B and C; oZIKV_2k for D and E) at MOI 1 for CHLA and MOI 0.1 for Daoy. The cell viability was analyzed 5 days after infection. Each bar represents one biological replicate plotted with mean and standard deviation (n = 5). Significance determined by one-way ANOVA Tukey’s multiple-comparison test. ∗∗∗∗p < 0.0001, ∗∗∗p < 0.001, ∗∗p < 0.01, ∗p < 0.05.

Before *in vitro* and *in vivo* testing of modified ZIKV strains to confirm oncolytic effect and safety, we performed a functional analysis to validate the inhibition mechanism of the MRE inserted in the ZIKV genome. As described in Figure 4A, the incorporation of MRE aimed to decrease ZIKV off-target toxicity in non-tumoral tissues by inhibiting virus replication in specific cells with a high miRNA expression.

To mimic the inhibition mechanism designed in the technology (Figure 4A), we overexpressed the corresponding miRNA in Daoy and CHLA06 cell lines before infection with oZIKV_2k and oZIKV_3′. Figures 4B and 4C show that miR-129-5p overexpression presented a tumor-suppressive role in both Daoy and CHLA06 by decreasing cell viability. However, the suppressive role of miR-129-5p is lost in the cell lines after oZIKV_3′ infection, indicating that the virus is being targeted and acts as an miRNA sponge.^26^ Most importantly, oZIKV_3’s oncolytic effects were reverted by miR-129-5p overexpression in Daoy, confirming the inhibitory mechanism of the technology (Figure 4C). The same was observed on oZIKV_2k, but with a higher viability increment after miR-219a-2-3p overexpression in both cell lines infected with oZIKV_2k (Figures 4D and 4E). This result confirms that the miRNA inhibition mechanism is working in the modified oZIKV produced.

### Cytotoxicity effect of oZIKV_2k and oZIKV_3′ in tumoral cell lines

**Figure 5:**
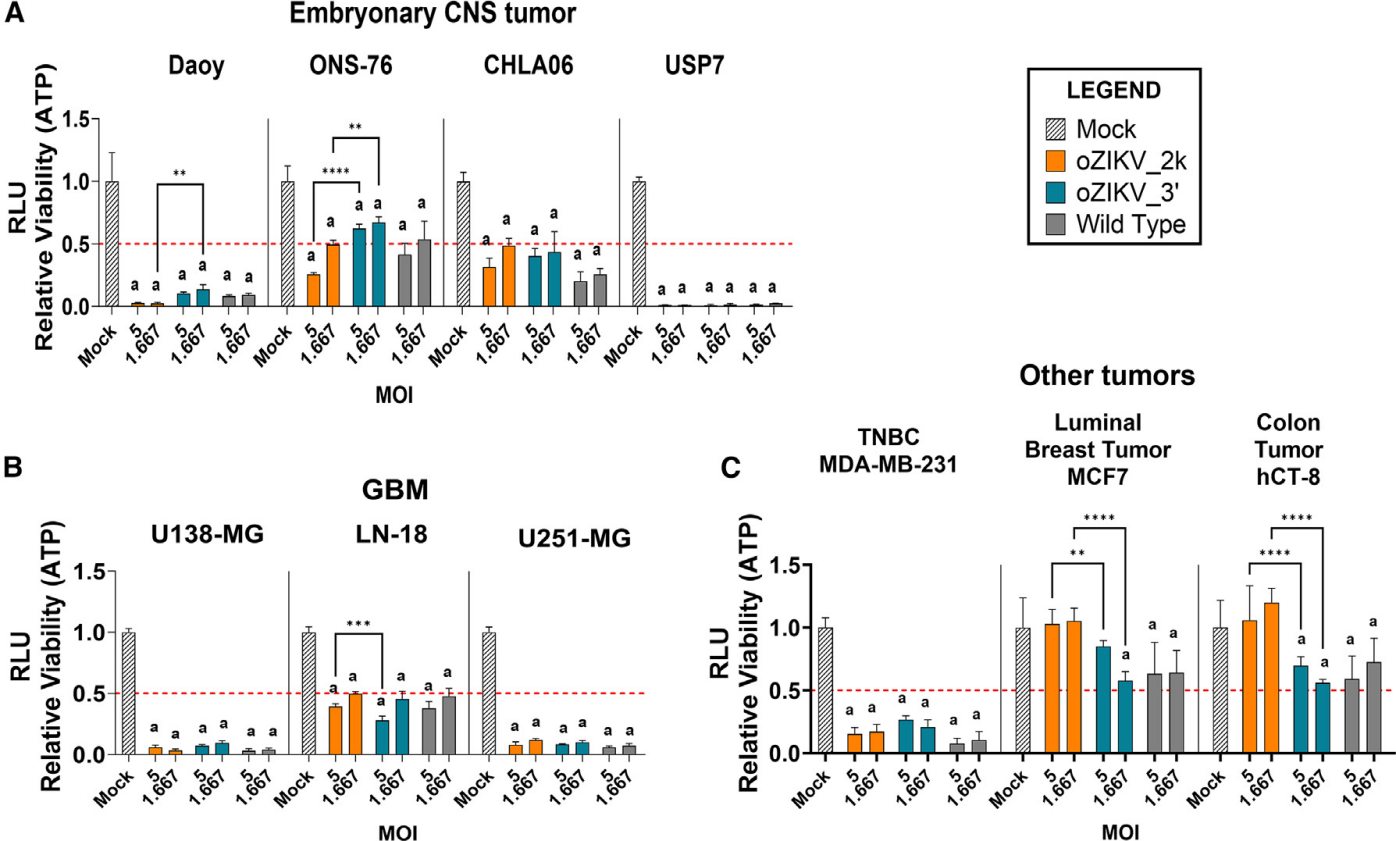
*In vitro* cytotoxicity effect of oZIKV_2k and oZIKV_3′ In (A)–(C), we report the oncolytic effect of oZIKV_2k, oZIKV_3′, and wild-type ZIKV at MOIs 5 and 1.667 in embryonal CNS tumor (A), glioblastoma (B), and other tumoral (C) cell lines by cell viability assessed 3 days after oZIKV_2k, oZIKV_3′, and wild-type ZIKV infection. For (A)–(C), each bar represents one biological replicate plotted with mean and standard deviation (n = 5). Significant difference among means was determined by one-way ANOVA Tukey’s multiple-comparison test. a = p < 0.0001 when compared with the Mock and ∗∗∗∗p < 0.0001, ∗∗p < 0.01, and ∗p < 0.05 when all groups were compared with all groups.

Once we had successfully produced the miRNA-sensitive ZIKVs and confirmed their inhibition mechanism, we went on to test the oncolytic effect in different tumor cell types. To do this we performed a cell viability assay after infection within a virus concentration curve of MOI 0.002, 0.007, 0.021, 0.062, 0.185, 0.556, 1.667, and 5 with oZIKV_2k, oZIKV_3′, and the ZIKV wild type (Figures 5 and S2). Figure 5A shows that both modified ZIKV significantly decreased cell viability in embryonal CNS tumor cells (Daoy, ONS-76, CHLA06, and USP07). The oncolytic effect observed was similar to the ZIKV wild type, except for oZIKV_3′ infection in the ONS-76 cell line, showing a viability decrease of about 50% in the highest MOIs, 30% less in comparison with oZIKV_2k and ZIKV wild type (Figures 5A and S2A–S2D). In the glioblastoma cell lines (U138-MG, LN-18, and U251-MG), the most aggressive CNS tumor in adults, both modified ZIKV strains retained the oncolytic effect compared with wild-type ZIKV (Figures 5B and S2E–S2G). When tested in the non-CNS tumor types of the triple-negative breast cancer (TNBC) cell line (MDA-MB-231), luminal breast tumor (MCF7), and colon tumor (hCT-8), the modified ZIKV effect was similar to the ZIKV wild type (Figures 5C and S2H–S2J). In the TNBC cell line, cell viability was significantly decreased after virus infection, evidencing therapeutic potential of oZIKV_2k and oZIKV_3′ for this breast cancer subtype.

### *In vitro* safety of oZIKV_2k and oZIKV_3′ in non-tumoral cell lines

**Figure 6:**
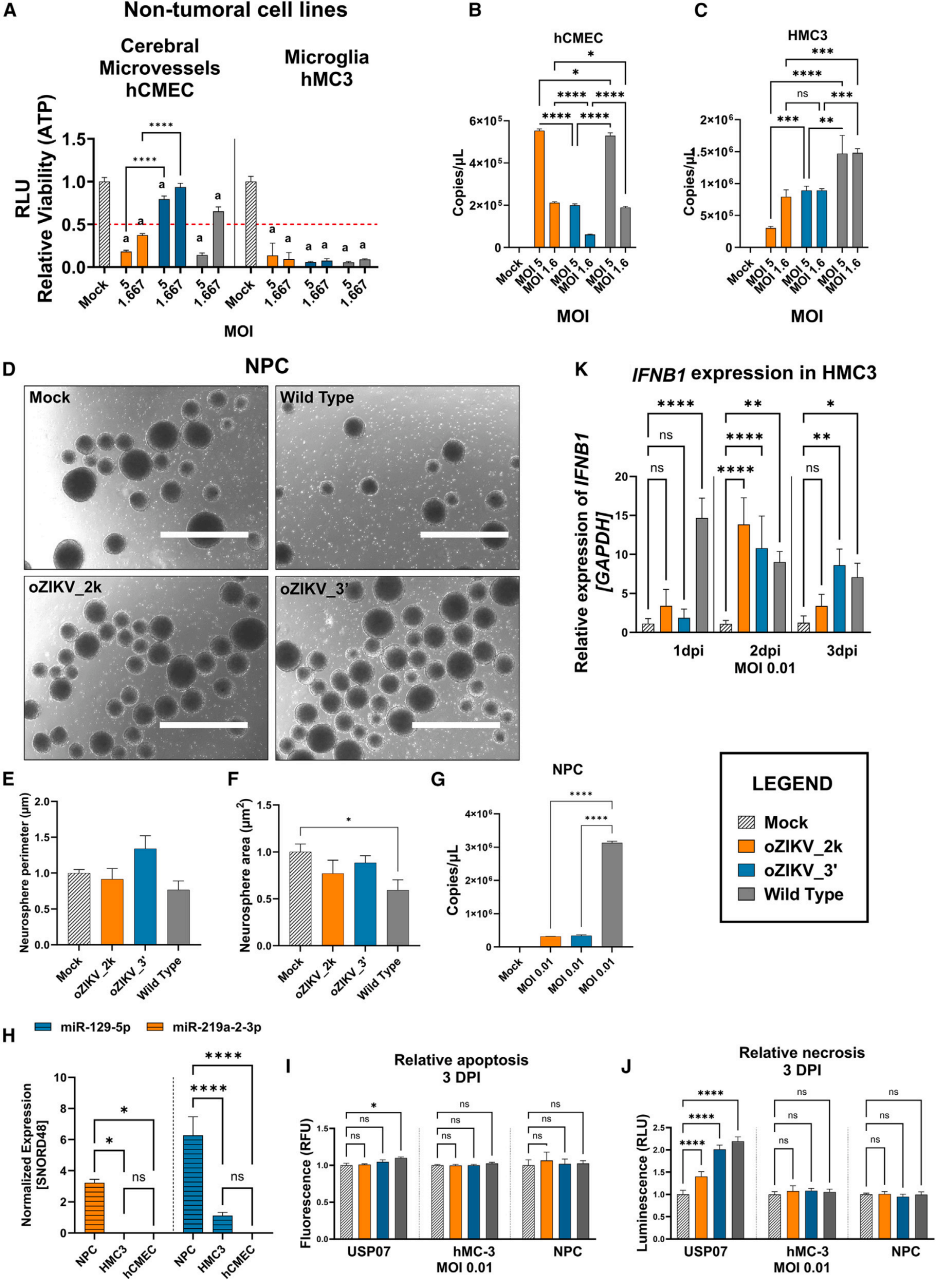
*In vitro* safety of oZIKV_2k and oZIKV_3′ In (A), the viability at 3 DPI of non-tumoral cells hCMEC and HMC3 after oZIKV_2k, oZIKV_3′, and wild-type ZIKV infection. Each bar represents one biological replicate plotted with mean and standard deviation (n = 5). Significant difference among means was determined by one-way ANOVA Tukey’s multiple-comparison test. *a* = p < 0.0001 when compared with the Mock and ∗∗∗∗p < 0.0001, ∗∗p < 0.01, and ∗p < 0.05 when all groups were compared with all groups. In (B) and (C), viral RNA copy quantification by RT-PCR of non-tumoral culture supernatant 3 days after oZIKV_2k, oZIKV_3′ and wild-type ZIKV infection. Each bar represents one biological replicate plotted with mean and standard deviation (n = 3). In (D), representative images of NPC neurosphere formation 5 days after oZIKV_2k, oZIKV_3′, and wild-type ZIKV infection at MOI 0.01. NPCs were differentiated for human iPS isolated from a patient with Congenital ZIKA Syndrome. Scale bar, 1000 μm. In (E) and (F), the neurosphere perimeter and area were quantified using the ImageJ program. Each bar represents one biological replicate plotted with mean and standard deviation (n of spheres = 100). (G) Viral RNA copy quantification by RT-PCR of neurosphere culture supernatant 5 days after oZIKV_2k, oZIKV_3′, and wild-type ZIKV infection. Each bar represents one biological replicate plotted with mean and standard deviation (n = 3). In (H), endogenous expression of miR-219a-2-3p and miR129-5p in non-tumoral (microglia, cerebral microvessel, and NPC) cell lines. Expression was normalized to SNORD48. Each bar represents one biological replicate plotted with mean and standard deviation (n = 3). In (I), apoptosis and (J), necrosis at 3DPI MOI 0.01 of non-tumoral cells hCMEC and HMC3 and tumoral cell USP07 as positive control of death after oZIKV_2k, oZIKV_3′, and wild-type ZIKV infection. Each bar represents one biological replicate plotted with mean and standard deviation (n = 4). (K) The normalized expression (*GAPDH*) of *IFNB1* in HMC3 cell line. Each bar represents one biological replicate plotted with mean and standard deviation (n = 3). For (A)–(F) and (H)–(K), significance was determined by one-way ANOVA Tukey’s multiple-comparison test. ∗∗∗∗p < 0.0001, ∗∗∗p < 0.001, ∗∗p < 0.01 ∗p < 0.05.

To address safety, we analyzed two non-tumoral cell lines, the cerebral microvessel hCMEC and microglia HMC3. Figures 6A and S2K show that oZIKV_2k infection decreased hCMEC cell viability below 50% at the two highest MOIs, as did wild-type ZIKV at MOI 5. The modified virus oZIKV_3′ showed lower cytotoxic effect and did not decrease hCMEC cell viability below 50% in any MOI. Indeed, the viral RNA levels of oZIKV_2k and wild type are similar and higher than found in oZIKV_3′ at the respective MOIs 5 and 1.66 (Figure 6B). In the HMC3 cell line, each ZIKV strain behaved similarly, leading to MOI-dependent decreased cell viability (Figures 6A and S2L), although fewer viral RNA copies were found after modified ZIKV infection when compared with wild type (Figure 6C).

We further analyzed the virus safety in NPC cells, a ZIKV known target (Figures 6D–6G). NPC neurospheres (NEs) were infected with oZIKV_2k, oZIKV_3′, and ZIKV wild type at MOI 0.01 (Figure 6D). Five days after virus infection, we quantified the NE perimeter and area (Figures 6E and 6F). Figure 6F shows that NE area significantly decreased after infection with ZIKV wild type, but not with oZIKV_2k and oZIKV_3′. We quantified viral RNA by RT-PCR after infection in NPC NE (Figure 6G) due to endogenous expression of miR-219a-2-3p and miR-129-5p, targeting MRE sequences of oZIKV_2k and oZIKV_3′, respectively, being higher in NPC than in other non-tumoral cells (Figure 6H). Indeed, both modified ZIKVs proved to be significantly less replicative than wild-type virus. These results indicate that both modified ZIKVs could exhibit improved safety in NPCs, based on virus replication inhibition when compared with the ZIKV wild type.

To better understand cell behavior after viral infection, we analyzed the cell death through apoptosis (Figure 6I) and necrosis (Figure 6J) assay in 2D culture of NPC, microglia, and USP07 tumor cell line used as positive control. Only the tumoral cell line (USP7) presented positive cell death after virus infection, especially necrosis, as previously reported by other groups.^8^ The non-tumoral cells, microglia, and NPC, do not show any kind of cell death after virus infection. To better understand the response of microglia after virus infection, we investigated the *IFNB1*, *IFNA1*, and *IFNA5* expression (Figures 6K and S3A–S3C) at the MOIs 0.1, 0.01, and 0.001, where the viability was near 50% after infections at 3 DPI. We found no expression of *IFNA1* and *IFNA5* (data not shown), and dose-dependent *IFNB1* expression after virus infection, indicating innate immune signaling activation.

### Oncolytic effect of oZIKV_2k in xenografic orthotopic model

**Figure 7:**
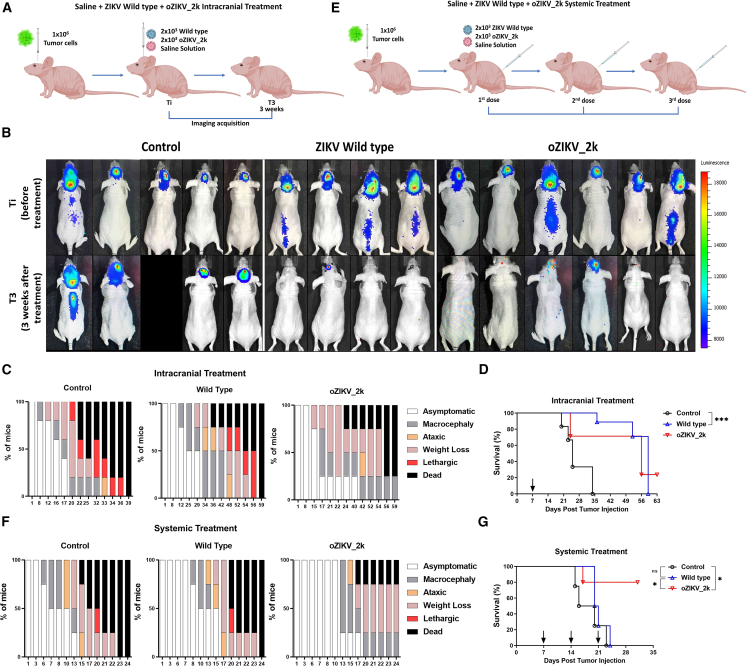
*In vivo* safety and tumor remission (A) Schematic representation of the *in vivo* experimental layout with a single intracerebroventricular virus administration at mice bearing intracranial human CNS tumor (USP7) used for (B), (C), and (D) data. (B) Representative bioluminescence-based images of tumor development in control (n = 5), wild-type ZIKV (n = 4), and oZIKV_2k (n = 6) treated mice. (C and F) Mice percentage presenting macrocephaly, ataxic, weight loss, lethargic and dead, during the time in days, after intracranial treatment (C) or systemic treatment (F) of control, wild-type ZIKV, and oZIKV_2k. (D and G), Overall survival rates of tumor-bearing mice after intracranial treatment (D) or systemic treatment (G). Significance determined by log rank Mantel–Cox test. ∗∗p < 0.01, ∗p < 0.05 ∗p < 0.05. (E) Schematic representation of the *in vivo* experimental layout with three systemic virus administrations on mice bearing intracranial human CNS tumor (USP7) used for (F) and (G) data.

The wild-type strain of Brazilian ZIKV infects and kills metastatic forms of human CNS tumors.^8^ Therefore, since oZIKV_2k showed cytotoxicity effect in CNS tumor cell lines (Figure 5), we tested whether oZIKV_2k presents equivalent oncolytic activity *in vivo* compared with the wild-type strain. Considering MRE mechanism, an *in silico* search at TargetScanHuman Release 8.0 using the mature miRNA sequence of hsa-miR-219a-2-3p (MIMAT0004675) showed dozens of conserved target sites among orthologs, including mice, strongly suggesting that human and mice share equivalent function of miR-219a-2-3p. To investigate oncolytic activity *in vivo*, we delivered 10^6^ USP7 cells in nude mice at the right lateral ventricle. After tumor engraftment (Ti), mice were divided into three groups, each group received one intracranial dose of 2 × 10^3^ PFU of wild type (n = 5–8), oZIKV_2k (n = 5–8), or saline as control (n = 5–8) (Figure 7A). As the wild-type strain, oZIKV_2k induced tumor remission in 83% (five of six) of treated animals and complete metastatic remission (tumors in the spinal cord) in 33% (two of six) of USP7 tumor-bearing mice (Figure 7B). Interestingly, oZIKV_2k treatment caused reduced clinical symptoms, such as ataxia and lethargy, when compared with the wild-type and control group, indicating fewer signs of tumor growth (Figure 7C). USP7 cells formed a very aggressive tumor in our experimental model, with a 100% death rate in saline-treated controls within 34 days. However, despite survival improvement in treated groups up to 60 days, there was no statistical significance between the wild-type and oZIKV_2k group (Figure 7D). Because ZIKV has a tropism for CNS neural stem-like cells and embryonal CNS tumors^8^^,^^10^ we tested the effectiveness of a different route of administration. Here we injected 10^6^ USP7 cells into the right lateral ventricle. After tumor establishment, mice received three doses of wild type, oZIKV_2k (2 × 10^3^ PFU), or saline intraperitoneally every 7 days (Figure 7E). Compared with intracranial treatment, which generated similar results among treated groups, oZIKV_2k did not generate symptoms until day 13 following peritoneal treatment (Figure 7F). Moreover, oZIKV_2k improved overall survival (p = 0.0459) over wild-type treatment with an 80% survival rate, while mice treated with wild-type virus, presented similar results compared with control group, as demonstrated on Figure 7G. When wild type and oZIKV_2k were intraperitoneally administered (2 × 10^5^ PFU) in Balb/C Nude tumor-free mice, both viruses were neutralized at serum 5 days after injection (Figure S4).

**Figure 8:**
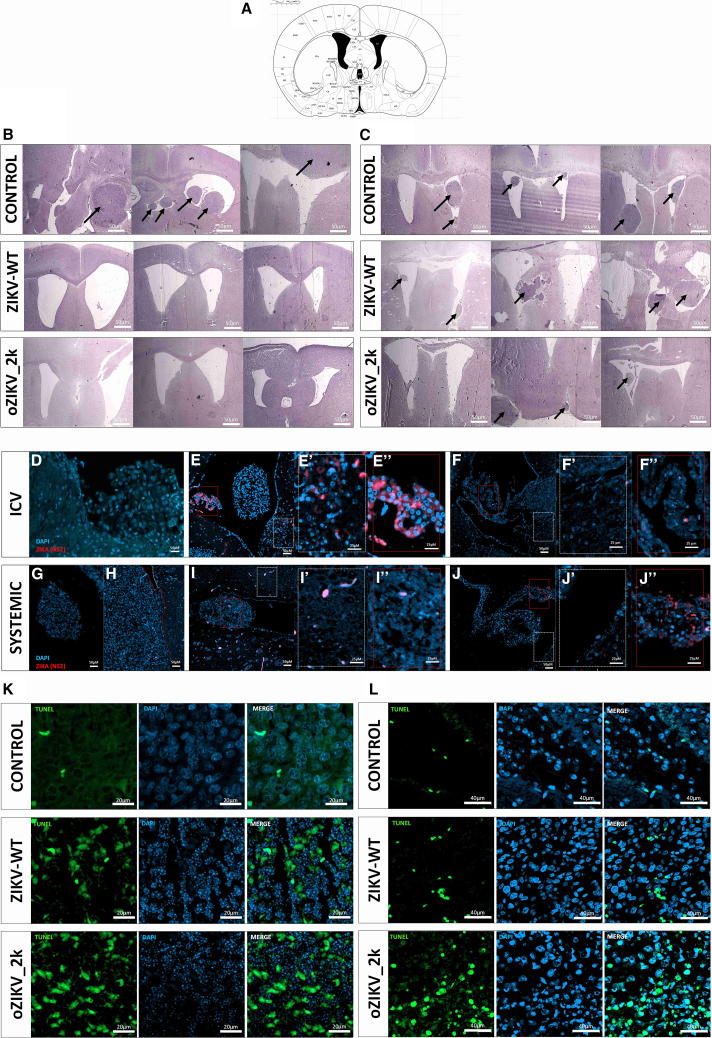
Histological images of brain tumor tissue Brain histopathology of tumor-bearing mice after intracranial treatment (B, D–E, and K) or systemic treatment (C, G–J, and L). In (A), coronal view of mouse brain showing the location of the coronal cut sections bregma 0.50 mm. Atlas templates were adapted from Paxinos and Watson (1998).^27^ In (B) and (C), representative images of hematoxylin and eosin-stained xenograft tumors. The black arrows indicate tumors in the different brain regions of mice. Scale bar, 50 μm. In (D)–(J), tissues show immunofluorescence labeling for NS2-ZIKV (red) and nuclei DAPI (blue). Scale bar, 50 μm. (D), (G), and (H) are the control groups. In (H), non-tumoral tissue is delineated by a dotted white line, while the tumoral tissue, characterized by a high density of large nuclei, is demarcated by a dotted red line. (E'), (F'), (I'), and (J') show non-tumoral tissue zoom from the white dotted square of (E), (F), (I), and (J). (E''), (F''), (I''), and (J'') show the tumoral tissue zoom from the red dotted square of (E), (F), (I), and (J). Scale bar, 25 μm. In (K) and (L), cell death staining by TUNEL assay (green), and nuclei DAPI (blue). In (K), scale bar, 20 μm. In (L), scale bar, 40 μm.

We further analyzed the brain tissue. H&E staining from brain coronal slices anteroposterior stereotaxic coordinates highlighted the difference between intracranial and systemic administrations (Figures 8A–8C, S5, and S6). The brain images showed less tissue damage following the systemic administration, with more preserved brain structure. No remaining tumor tissue was found in the group treated with intracranial injection of both ZIKV wild type and oZIKV_2k after screening all H&E brain slices (Figures 8B and S5). In contrast, smaller remaining tumor tissue was found in all mice treated with oZIKV_2k when compared with ZIKV wild-type and control group after systemic virus administration (Figures 8C and S6). Immunofluorescence staining of ZIKV NS2, cell death (TUNEL), and the nuclei (DAPI) showed positive cell death and ZIKV staining at the tumor mass in all groups treated with oZIKV_2k and ZIKV_WT, including the intraperitoneal injection group, confirming the modified virus CNS tropism and capability of crossing the BBB and *in vivo* oncolytic effect (Figures 8D–8L). In the ZIKV-WT treated group, ZIKV staining was detected in both the tumor mass (Figures 8E" and 8I") and mice tissue (Figures 8E' and 8I'). Conversely, non-tumoral mouse tissue in the oZIKV_2k systemic treated group exhibited no positive staining of the NS-2 ZIKV protein (Figure 8j').

Altogether, these data suggest that oZIKV_2k presents equivalent CNS tropism to the wild-type strain, targets tumor cells, exhibits fewer side effects, and improves survival in mice, indicating peritoneal administration and multiple doses of low virus load as an efficient and safer alternative route to intracranial treatment.

## Discussion

Through genetic engineering of the Latin American epidemic virus ZIKV, we have generated a first-in-class miRNA-sensitive oncolytic virus with CNS tropism and low infectivity in the neural progenitor cells whose infection has been associated with microcephaly in babies.^28^^,^^29^ For the first time, an active ZIKV was developed with a tiny non-random modification in the RNA genome, which did not attenuate the virus replication/infectability, and made it more selective to cancer cells compared with the wild-type strain.

This breakthrough technology is based on systemic administration and offers great treatment promise for patients with malignant CNS brain tumor, compared with previous drug and immunotherapy strategies, which have failed to overcome factors including the BBB and immune-cold tumor microenvironment.^30^

Compared with the other oncolytic viruses, the ZIKV virus offers unique advantages for oncolytic application, including targeting (tropism),^11^ mechanism of action (cytotoxicity in cancer cells and immune action),^31^ and pharmacodynamics (rapid clearance and barriers to safety).^32^ The miR-sensitive oZIKV developed here kept all these features, combined with reduced replicative capability in NPCs, mitigating the predicted risk of microcephaly.^29^ Herpes simplex virus (HSV), the first virotherapy to be approved by the FDA, has low pathogenicity but required extensive genetic modification to preferentially target cancer cells and to enhance immunogenicity (e.g., incorporation of a GM-CSF transgene).^33^ Likewise, adenovirus vectors lack key features required for a successful oncolysis, necessitating genetic modification—addressing for example intracellular oncogenic pathways, immune-avoidance mechanisms, extracellular oncogenic receptors, and modified promoters.^34^ Other less common viruses, like newcastle virus, vaccine virus, measles, and poliovirus are naturally oncolytic, but have limited CNS specificity and immunogenicity.^31^ Oncolytic viruses have been engineered over the past 2 decades aiming to use miRNA-based control of gene expression to improve safety, reduce toxicity, and enhance tropism to tumoral cells without losing oncolytic effect by viral attenuation.^35^ While there has been success in employing recombinant viruses tailored to the host’s miRNAs with cell-type-specific responses, the Zika virus, as of now, has not undergone engineering to include an MRE insertion that can efficiently diminish viral replication in NPC cells without compromising oncolytic efficacy.

An additional innovative aspect of this technology relates to the MRE insertion sites. The UTR sequences of flavivirus are very sensitive to modifications since the subgenomic flaviviral RNA (sfRNA), a functional RNA derived from viral 3′UTR, plays a crucial role in virus replication and pathogenesis by inhibiting host cell anti-viral responses.^36^ Other studies have shown that a single nucleotide modification in the UTRs can lead to virus attenuation and loss of infectivity.^37^ Therefore, to preclude virus replication in normal tissue, preserve oncolytic effect, and minimize insertion of additional genetic material, we used an established miRNA inhibition approach to improve virus selectivity.^17^ However, we have employed insertion sites in the ZIKV genome that have never previously been reported—in the middle of the coding RNA and just before 3′UTR. Innate immune signaling activation of IFNβ, which drives JAK/STAT signaling, was observed after virus infection in myeloid cells.^12^ Cytotoxicity of miRNA-sensitive ZIKVs was demonstrated after infecting embryonal CNS tumor, glioblastoma, and, in an unrelated tumor model, we showed that TNBC can also be a target of the oZIKV. Previous data generated by our research group and others showed that a cell line derived from luminal breast cancer (MCF-7) was resistant to the ZIKV oncolytic effect.^8^ The challenge of breast cancer is the patients bearing triple-negative tumors who do not respond to chemotherapy, succumbing to the disease.^38^ These TNBCs are also known to express stem-like receptors and present a subpopulation of cancer stem cells that are responsible for generating metastasis, therapy resistance, and tumor remission.^39^^,^^40^ Remarkably, the ZIKV has a selectivity for stem-like cell lines, showing a tropism for cell-expressing stem cells markers like SOX2.^8^^,^^10^^,^^13^

Therefore, safety is a primary concern for this therapy approach, since it is based on a replication-competent pathogenic virus. Administration of oZIKV_2k to immunosuppressed mice provided encouraging safety data, since the increase of drug toxicity by systemic administration of three doses highlighted not only the CNS tropism but also revealed a survival difference between the oZIKV_2k and the wild-type group.

Systemic administration of ZIKV is a great innovative feature of the present therapy for CNS tumors. It is possible since Zika virus presents a natural tropism to CNS^41^ and is also a less invasive alternative route of administration. Most oncolytic therapies with clinical trial approval preferred intratumoral administration, including the G47Δ^3^, which maximizes the viral load in the tumor, but this is challenging in the context of CNS tumors.^42^

For systemic administration there is a need to overcome viral neutralization, organ sequestration, and vessel extravasation, to achieve tumor cell death. Nevertheless, a phase II clinical trial of oncolytic virus H101 was conducted to evaluate the effectiveness and safety of intraperitoneal injections in patients and may become a novel route of oncolytic therapies (clinicaltrials.gov, NCT04771676). However, the animal model used here is an immunodeficient mouse without thymus, and more studies need to be done with a more complex animal model to confirm the systemic administration efficiency and safety. The natural history of ZIKV infection is benign: 80% of infected adults are asymptomatic, clinical manifestations are mild and self-limiting, and the severe complications are associated with the infection of NPCs that are present only in the early stages of fetus development. Therefore oZIKV_2k has great potential as an oncolytic therapy against CNS tumors, especially because the *in vitro* data showed that the candidate strain is not cytotoxic and does not replicate effectively in NPC cells, the main virus target. In sum, the miRNA-sensitive oncolytic ZIKV presented offers real promise as an effective therapy for incurable CNS tumors, an unmet disease responsible for about 250,000 deaths per year all over the world.^43^

## Materials and methods

### Cell culture

The commercial cell lines were purchased and cultivated according to American Type Culture Collection (ATCC) or Rio de Janeiro Cell Bank (RJCB) recommendations. Donated cell lines were cultivated according to the correspondent reference. The cell lines were as follows: Medulloblastoma DAOY (ATCC HTB-186), ONS-76 (BCRJ 0294), and USP13-MED (in-house established^44^ kindly donated by Human Genome and Stem Cell Research Center [HUG-CELL]); Atypical Teratoid/Rhabdoid Tumors CHLA-06-ATRT (ATCC CRL-3038) and USP07-ATRT (in-house established^8^ kindly donated by HUG-CELL); Glioblastoma LN-18 (ATCC CRL-2610), U251-MG (Sigma Aldrich/09063001, donated by HUG-CELL), and U138-MG (ATCC HTB-16); Breast Cancers MCF7 (BCRJ 0162) and MDA-MB-231 (BCRJ 0164); Colorectal Cancer hCT-8 (BCRJ 0106); Microglia non-tumoral HMC3 (ATCC CRL-3304); and Cerebral microvessel non-tumoral hCMEC/D3 (BCRJ 0401). Cell lines were maintained up to 8 weeks (passages 1–16) and Tryple Select (Thermo Fisher Scientific) was used in routine subculture for cell dissociation. A commercial NPC line (Lonza-discontinued) and two human-induced pluripotent stem cell-derived NPCs (hiPSC) (CH/C2 and 763-1, in-house reprogrammed^18^) were kindly donated by HUG-CELL. NPCs were maintained in DMEM/F12 (Thermo Fisher Scientific), N2 1x (Gibco), B27 1x minus vitamin A (Gibco), 10 μg/mL EGF (Gibco), 10 μg/mL FGF (Gibco). Monolayer NPC culture was coated in hESC-Qualified matrigel (Corning). Three-dimensional spheroid NPC culture was maintained in low attachment plates (Corning) under 91 RPM rotation. Accutase (Thermo Fisher Scientific) was used in routine NPC subculture or sphere dissociation up to passage 9. All cell lines were maintained with Normocin - Antimicrobial Reagent (InvivoGen) 100 μg/mL and incubated at 37°C, 5% CO_2_ in air atmosphere. Cells were tested for *Mycoplasma* contamination by MycoAlert PLUS-Mycoplasma Detection Kit (Lonza LT07-710) before use in the described experiments. Cell authentication was performed by short tandem repeat genotyping by Biospot Ltda-SP, Brazil.

### MiRNA expression profile

Small RNAs were isolated using phenol-chloroform-based mirVana miRNA Isolation Kit (Invitrogen). Samples were quantified by NanoDrop Spectrophotometer (Thermo Fischer Scientific), the RNA purity was evaluated by the ratio 260/280 nm, and 10 ng was submitted to cDNA synthesis reaction with TaqManAdvanced miRNA Assay Kit (Applied Biosystems). TaqMan Fast Advanced Master Mix (Applied Biosystems) and TaqMan Advanced miRNA Assays 477979_mir, 477980_mir, 477896_mir, 478886_mir, and Hs04931161_g1 were used for detection of mature miRNAs has-miR-219a-2-3p, has-miR-219a-5p, has-miR-129-5p, has-miR-4298, and the control small nucleolar RNA C/D box 48, respectively, by RT-PCR amplification with QuantStudio 7 Flex. Reactions were conducted according to the respective manufacturer protocols. RT-PCR quantification was based on linear regression analysis from standard curves with amplification efficiency ranging from 90% to 100%.

### miRNA modulation of modified oZIKVs and PCZ

The oligonucleotides mirVana miRNA mimic hsa-miR-129-5p (assay ID MC10195) and hsa-miR-219a-2-3p (assay ID MC11150) were used for mature miRNA expression, and mirVana miRNA Mimic Negative Control #1 was used as a control. The transfection mix was prepared with 250 μL/mL of Opti-MEM, 50 nM of desired oligonucleotide, and 2.5 μL/mL of transfection reagent Lipofectamine MessengerMAX following the steps of the manufacturer’s protocol. Cell lines to be transfected were detached and resuspended in the routine cell media at 2 × 10^4^ cells/mL. Each transfection reaction was carefully mixed to 1 mL of cell suspension, and this was transferred to a 96-well culture plate at final volume of 100 μL/well. Plates were incubated for 24 h. After that, for PCZ modulation, cell media was removed and another transfection was performed directly in the 96-well plate, without detaching cells, using the same transfection mix reagents but containing 2.5 μg of PCZ of interest instead of miRNA oligonucleotide. Cell viability was analyzed the next day. For modified oZIKV modulation, after 24 h of miRNA oligonucleotide transfection, viruses were defrosted and maintained at 4°C–10°C during the cell media removal of plates. Cells were infected with 20 μL of the modified virus of interest diluted in cold Opti-MEM at MOI 0.1 for Daoy infections and MOI 1 for CHLA06 infections. After infected plates were incubated for 30 min, 200 μL of complete cell media was added to each well. Mock cells were incubated with only Opti-MEM. Cell viability was assessed after 5 days.

### Molecular clone design of ZIKV

The parental plasmids PCZ and oZIKV were synthesized by GenScript. For the insertion of the MRE candidates in PCZ, a BsiWI cloning site was inserted in place of previous HA sequence, as described in Figure S2. Primers set with the MRE flanked by the cloning site were annealed by heating to the melting temperature and cooled to room temperature (Figure 2B). The product was phosphorylated and cloned with T4 ligase into the previously digested PCZ plasmid. After ligation overnight at 4°C, the plasmid was transformed into DH10B competent cells by electroporation, plated, and colonies formed were propagated in liquid media. Miniprep was performed using GeneJET Plasmid Miniprep Kit by Thermo Scientific. Purified plasmids were analyzed by Sanger sequencing for cloning diagnosis. For cloning the MRE targets in oZIKV, the same strategy was used for insertion of miR-129-5p MRE into the MluI cloning site in oZIKV_3′ and miR-219a-2-3p MRE into the NsiI cloning site for oZIKV_2k.

### *In vitro* transcription

The PCZ constructions were linearized by Mlu/EcoRI and oZIKV by NotI, then purified by precipitation with ethanol and 10% ammonium acetate (3M) and resuspended in 8 μL ultrapure water. This linearized DNA was transcribed using MEGAscript T7 Transcription Kit, the reaction was incubated at 37°C for 2 h, and TURBO DNase was added for 15 min. The synthetic RNA was precipitated with 30% volume LiCl and 2x volume isopropyl alcohol overnight. The RNA was pelleted by centrifugation at 15,000 × *g*, washed with 70% ethanol, and resuspended with RNAse free water.

### Cell transfection

The synthetic RNA was quantified using NanoDrop Spectrophotometer (Thermo Fischer Scientific), the RNA purity was evaluated by the ratio 260/280 nm. Then, 2.5 μg of transcribed RNA was electroporated into 1 × 10^6^ Vero Cells in 4-mm cuvettes with the GenePulserXcell (Bio-Rad) at settings of 200 V and 960 μF, pulsing one time. After electroporation, the transfected cells were seeded in a T-25 flask with 12 mL of DMEM High medium (Gibco) supplemented with 5% fetal bovine serum and normocin (100 mg/mL) followed by incubation at 37°C and 5% CO.^2^ The cells were monitored daily for cytopathic effect. Supernatant was harvested at 5 days post-transfection, clarified by centrifugation at 400 × *g*, and stored in aliquots at −80°C. Viral recovery was confirmed by RT-PCR, and the integrity of the genome of PZC was confirmed by nucleotide sequencing.

### Cell viability assay

The CellTiter-Glo Luminescent Cell Viability Assay was used to detect metabolic active cells by quantifying ATP levels. Cells were seeded at 4 × 10^3^ cells/well in 96-well culture plates at final volume of 100 μL/well 1 day before the infection. In the moment of infection viruses were defrosted and maintained at 4°C–10°C during the preparation of a serial dilution of 8 points with dilution factor of 1:3 starting with MOI 5, followed by MOIs 1.667, 0.556, 0.185, 0.062, 0.021, 0.007, and 0.002. All points of this MOI curve were diluted in cold Opti-MEM. After dilutions were made, cell media was removed, and five-well replicates of each cell line were incubated with 20 μL of each MOI for 30 min. Mock cells were incubated with only Opti-MEM. After incubation, 200 μL of complete cell media was added, and plates were incubated for 3 days. To evaluate cell viability of previously transfected or infected plates, the kit’s reagents were prepared, acclimatized at room temperature, and reactions were proceeded according to the manufacturer’s protocol. Reactions were transferred to opaque white multiwell plates, and the luminescent signal was acquired by luminometer (GloMax Discover Microplate Reader, Promega).

### Cell death assay

The RealTime-Glo Annexin V Apoptosis and Necrosis Assay was used to measure the cell death process. HMC3 and USP07 cells were seeded at 4 × 10^3^ cells/well in white-clear bottom 96-well culture-treated plates. NPC cells were seeded at 2 × 10^4^ cells/well in matrigel-coated wells. Cells were infected with 20 μL oZIKV_2k, oZIKV_3′, and wild type at MOI 0.01 for 30 min. Mock cells were incubated with Opti-MEM. After incubation, 100 μL of complete cell media was added, and plates were incubated until the next day. At 1 DPI, cells were treated with mixed reagents of the RealTime-Glo Annexin V Apoptosis and Necrosis Assay according to manufacturer’s instructions. The endpoint luminescence and green fluorescence (485 nmEx/525–530 nmEm) were acquired by luminometer (GloMax Discover Microplate Reader, Promega) at 3 DPI.

### Viral RNA titer: RT-PCR

ZIKV RNA obtained from culture supernatants was extracted by using Viral RNA Mini Kit (Qiagen), according to the manufacturer’s protocol and viral RNA copies were quantified by RT-qPCR. For absolute RT-qPCR titration, a standard curve with six dilutions was generated with a double-strand DNA fragment with a sequence corresponding to a region of Zika E protein. Samples were amplified along with standard curve dilutions, in three replicates each on a Stepone Real-Time PCR System and TaqMan Fast Virus 1-Step Master Mix (Thermo). The total Zika RNA copy number was calculated by multiplication of the cDNA copy number by a conversion factor, which considered all dilutions made during the RNA extraction to qPCR protocol. Primer sequences were CCGCTGCCCAACACAAG (Forward), CCACTAACGTTCTTTTGCAGACAT (Reverse), and 5′FAM AGCCTACCTTGACAAGCAGTCAGACACTCAA3′ BHQ1 (Probe).

### Virus titration

To determine the amount of infectious viral particles (PFU) Titration (in PFU mL^−1^) was obtained by plaque assay. *Cercopithecus aethiops* kidney epithelial Vero cells (ATCC- CCL81) were used for virus propagation and for plaque assays. Briefly, Vero cells (1 × 10^5^/well) were plated in 24-well culture plates (Sarstedt, Inc.) and incubated at 37°C in a CO_2_ incubator overnight. Viruses' supernatant aliquots (50 μL) were 10-fold serially diluted in medium, added to Vero cells, and incubated at 37°C for 1 h to allow virus adsorption. The viral supernatant was aspirated, and each well was overlayed with a prewarmed solution of 0.6% agarose (Thermo Fisher) and DMEM High medium (Gibco). After 4 days of incubation at 37°C, plaque visualization was made using blue-black staining solution. The most appropriate viral dilution was estimated to determine the number of infected cells visible (PFU mL^−1^). All the subculture aliquots were stored in cryovials and maintained in −80°C.

### Quantification of *IFN* by quantitative reverse-transcriptase PCR - qRT/PCR

Cells infected with oZIKV_2k, oZIKV_3′ and wild type at MOIs 0.1, 0.01, and 0.001 were harvested at 1 DPI, 2 DPI, and 3 DPI and lysate with RLT-BME Buffer. RNA was extracted with RNeasy Kit (Qiagen), according to the manufacturer’s protocol. The RNA reverse transcription was performed with SuperScript III and oligo-dT, and qPCR was carried out using TaqMan advanced master mix (applied biosystem, Thermo Fischer). TaqMan Gene Expression Assays primers were used for *IFNA1* (AssayID:Hs03044218_g1), *IFNA5* (AssayID:Hs04186137_sH), *IFNB1* (AssayID:Hs01077958_s1) and *GAPDH* (AssayID:Hs02786624_g1). Relative quantitation was based on 2^−ΔΔCt^ method.

### Mice and treatment groups

Six- to 8-week-old female Balb/C Nude mice were used in all experiments. The study followed the International Ethical Guideline for Biomedical Research (CIOMS/OMS, 1985) and was approved by the Institutional Animal Experimentation Ethics Committee (CEUA-USP no. 408/2023). A total of 57 animals were included in the present study. To analyze virus biodistribution by virus RNA quantification (RT-PCR) after systemic administration, 24 Balb/C Nude mice tumor-free were separated into wild type (n = 12) and oZIKV_2k (n = 12) for blood serum and tissue collection at 0, 3, 5, and 7 DPI, three mice per endpoint. Mice bearing CNS tumor cells were separated into wild-type (n = 15), oZIKV_2k (n = 15), and control saline group (n = 15). To minimize animal suffering, the following clinical symptoms were observed and monitored before euthanasia: 30% weight loss and/or ataxia and/or visible tumor and/or freezing. Animals were randomly divided into two groups for either intracranial or systemic treatment.

### Wild-type ZIKV and oZIKV_2k treatments in an orthotopic metastatic xenograft model

USP7 tumor cell lineage expressing luciferase was generated with pLV/Luc lentiviral vector, as previously described.^45^ Tumor injections and virus treatment were performed as previously described.^46^ Mice received 10^6^ cells into the right ventricle. After tumor establishment, 1 week after tumor injection, according to cell line-dependent growth kinetics, mice received 2 × 10^3^ PFU particles of wild-type ZIKV or oZIKV_2k, intracranially and peritoneally, one and three doses, respectively. Doses were given 1 week apart. The control group received intracranial or intraperitoneal PBS application. Tumor development was assessed *in vivo* with the IVIS Imaging System (PerkinElmer) as previously described.^44^ The animals were weighed every 2 days and symptoms were defined according to the following manifestations: macrocephaly, ataxia, and weight loss. After 30% weight loss, mice were euthanized.

### Biodistribution assay

To analyze the biodistribution of wild-type ZIKV or oZIKV_2k in tumor-free conditions, Balb/C Nude mice received a single dose of 2 × 10^5^ PFU/mL intraperitoneally. Blood serum, spleen, liver, and reproductive organs were harvested at 1, 3, 5, and 7 DPI and stored at −80°C. Frozen tissues were homogenized and virus RNA extraction and quantification were performed as described previously.

### Histopathology

Immediately after controlled euthanasia, all tissues were fixed with a 4% paraformaldehyde solution for 24 h at room temperature, and paraffin sections with 4-μm thickness were processed for H&E staining, immunofluorescence of ZIKV NS2 protein (GTX133308, Genetex, 1:500) and quantitation of apoptotic cells by DeadEnd Fluorometric TUNEL System (Promega). Sections were deparaffinized. For immunofluorescence, tissue sections were blocked and prepared as described by Kaid et al., 2020.^9^ For the TUNEL assay, we followed the fabricant’s instructions. All images were taken in a confocal microscope (Zeinss LSM 800).

## Data and code availability

The data that support the findings of this study are available within the article and its supplementary data files. The data not publicly are available on request from the corresponding author, C. Kaid.

## Supplemental information

Document S1. Figures S1–S6 and Table S1

Document S2. Article plus supplemental information
