# Supplementary material for: Genetically modified ZIKA virus as a microRNA-sensitive oncolytic virus against central nervous system tumors
Source: Mol Ther. 2024 Jan 11;32(2):440–56. doi: 10.1016/j.ymthe.2024.01.006 (PMC10861990; doi:10.1016/j.ymthe.2024.01.006)
Supplement: Document S1. Figures S1–S6 and Table S1 [file mmc1.pdf]

YMTHE, Volume *32*

Supplemental Information

Genetically modified ZIKA virus as a microRNA-sensitive oncolytic virus against central nervous system tumors

Gabriela Machado Novaes, Caroline Lima, Carla Longo, Pedro Henrique Machado, Thais Peron Silva, Giovanna Gonçalves de Oliveira Olberg, Diego Grando Módolo, Márcia Cristina Leite Pereira, Tiago Goss Santos, Mayana Zatz, David Lagares, Marcelo de Franco, Paulo Lee Ho, Harry Bulstrode, Oswaldo Keith Okamoto, and Carolini Kaid

Supplemental and additional information

Table S1: In-silico expression profile of miRNAs

**miRNA**	**Sequence**	**Accession Number***	**Positive expression in normal tissue****	**Downregulated in cancer vs normal*****
miR-4298	CUGGGACAGGA GGAGGAGGCAG	MIMAT0016852	Spleen, lymph node, brain and other tissues	Colorectal cancer, kidney cancer, cervical cancer, brain cancer and other tumors
hsa-miR-129-5p	CUUUUUGCGGU CUGGGCUUGC	MIMAT0000242	Brain, nerve, spinal cord and testis	Brain cancer, kidney cancer and hepatocellular carcinoma.
hsa-miR-219a-2-3p	AGAAUUGUGGC UGGACAUCUGU	MIMAT0004675	Brain, spinal cord and nerve	Brain cancer, thyroid cancer, gastric cancer, colorectal cancer and other tumors
hsa-miR-219a-5p	UGAUUGUCCAA ACGCAAUUCU	MIMAT0000276	Brain, nerve and spinal cord	Ovarian cancer, sarcoma, brain cancer, colorectal cancer and other tumors.
*** Based on mirBase.org database.**				
**** Tissues are in descending order of expression based on TissueAtlas- Human miRNA Patterns database (https://ccb-web.cs.uni-saarland.de/tissueatlas2/).**				
***** Cancer suppressor miRNA expression is in increasing order of expression based on a Database of Differentially Expressed miRNAs in Human Cancers (dbDEMC) (https://www.biosino.org/dbDEMC/index).**				

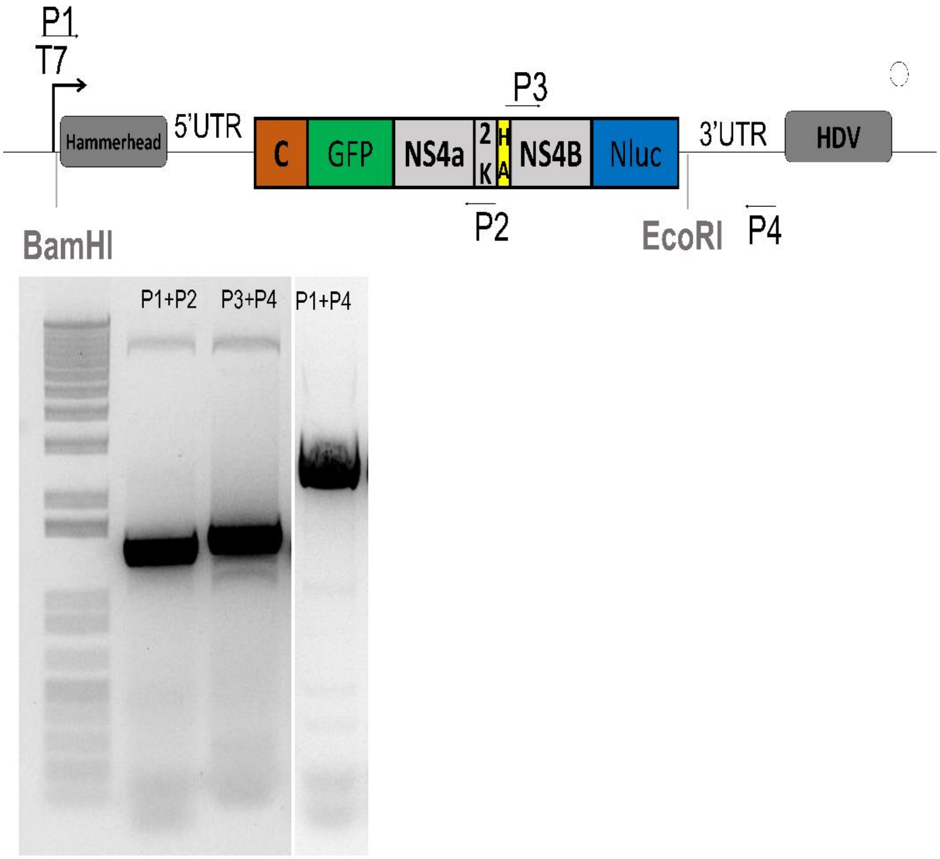

Fig.S1: Insertion of BsiWi cloning site in PCZ

For the insertion of BsiWI cloning site, a fusion PCR was performed where primers were designed to amplified parts of 2k and nucleotides corresponding to the BsiWI site. Set of primers P1 + P2 and P3 + P4 (Supplementary Figure 2) generated two fragments that were assembled in a second PCR using both fragments as templates and P1+P4 primers. This PCR product was digested with BamHI and EcoRI and cloned at the same sites in the first partial oZIKV plasmid.

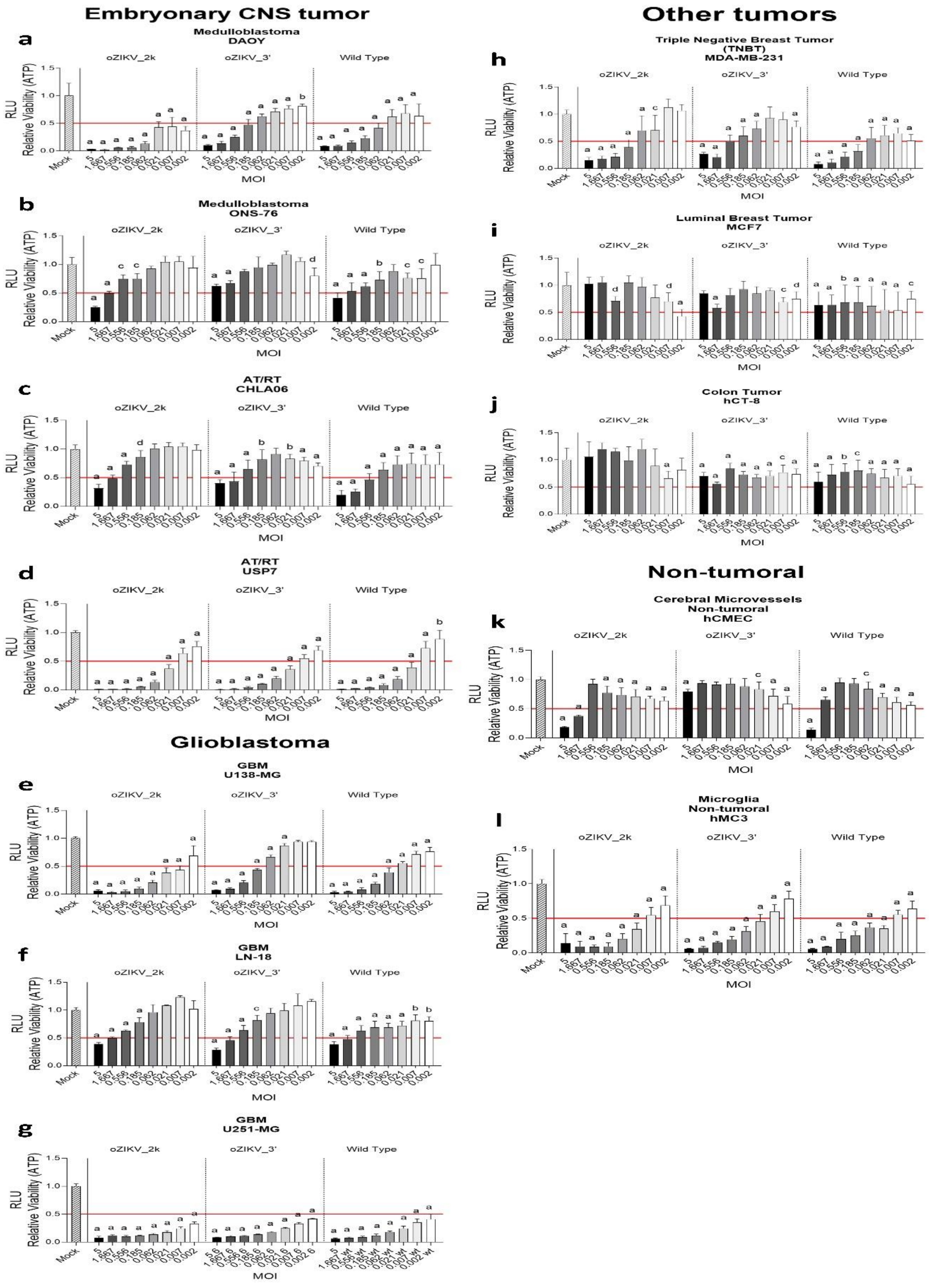

Fig.S2: In vitro cytotoxicity effect of oZIKV_2k and oZIKV_3'

In **a-k,** we report the effect of oZIKV_2k, oZIKV_3', and wild-type ZIKV infections on cell viability at MOIs 5, 1.667, 0.556, 0.185, 0.062, 0.021, 0.007 and 0.002 in embryonal CNS tumors (**a-d**), glioblastoma (**e-g**), other tumors (**h-j**) and non-tumoral cell lines (**k-l**). Cell

viability was assessed 3 days after infection. Each bar represents one biological replicate plotted with mean and standard deviation ($n = 5$). Significant difference among means was determined by One-way ANOVA Tukey's multiple-comparison test. Infections were compared with Mock and a significant difference was represented as *a*=$P < 0.0001$, *b*=$P < 0.001$, *c*=$P < 0.01$ and *d*=$P < 0.05$.

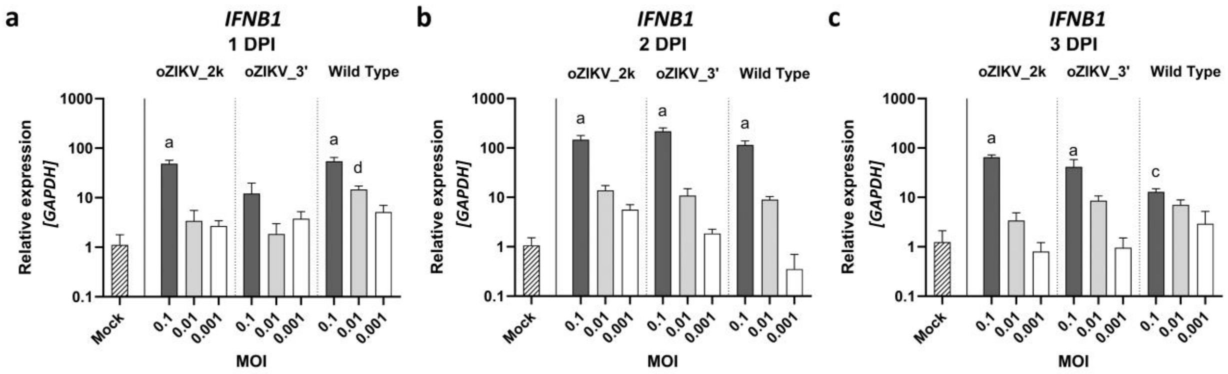

Fig.S3 – *IFB1* expression in microglia cell line

IFB1 expression analysis by TaqMan RT-PCR of microglia cell line (HMC3) after oZIKV_2k, oZIKV_3' and wild type infection after 1 (**a**), 2 (**b**), and 3 (**c**) days post-infection (DPI).

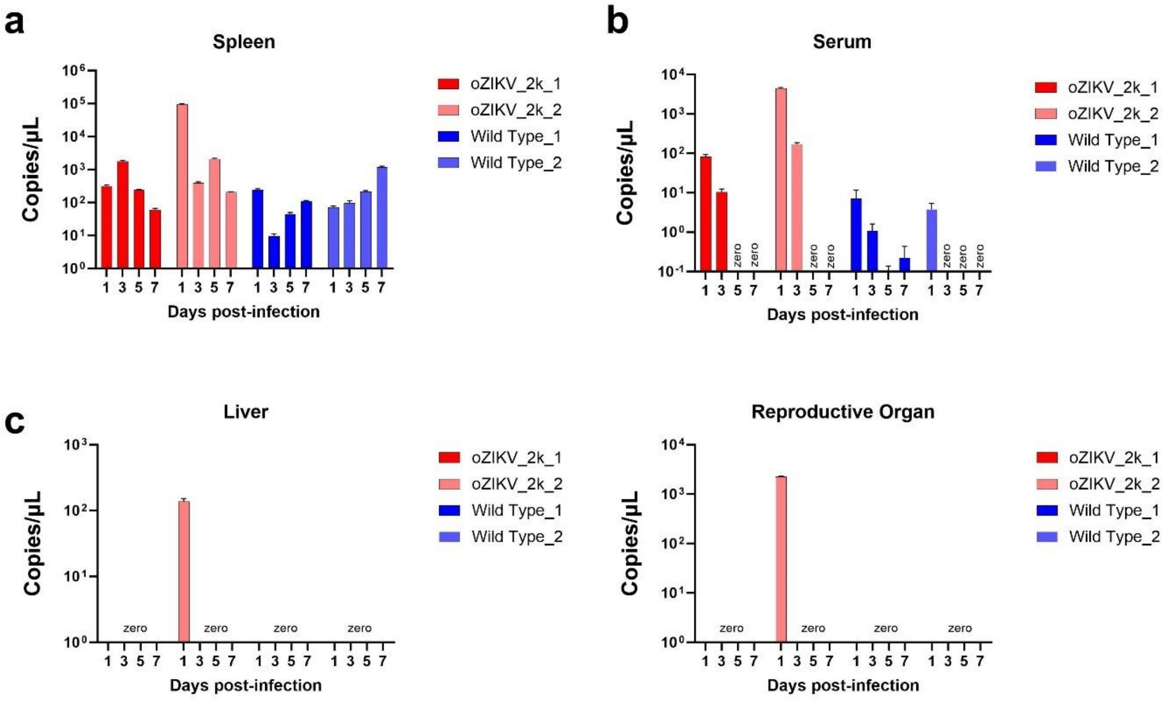

Fig.S4 - Biodistribution of wild-type ZIKV and oZIKV_2k in tumor free model

Viral RNA copy quantification by RT-PCR of **a**, spleen, **b**, serum, **c**, liver, and **d**, reproductive organ 1, 3, 5 and 7 days after oZIKV_2k, and wild-type ZIKV infection. Each bar represents one biological replicate plotted with mean and standard deviation (n = 3).

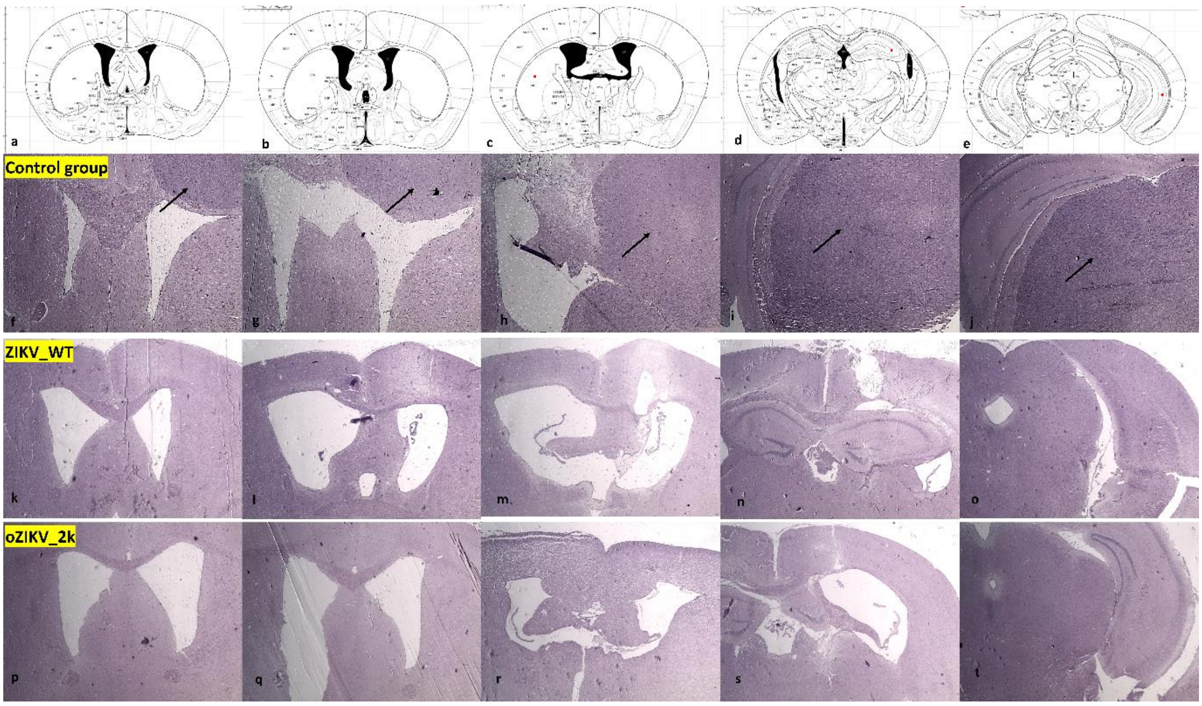

Fig.S5 - Histological images of brain tumor tissue after intracranial treatment

In **a-e,** brain coronal slices anteroposterior stereotaxic coordinates of the sections relative to bregma 0.50 and -7 mm. The **black arrows** indicate tumors in the different brain regions of mice. In **f, t**he tumor shows the right cortex. In **g**, the tumor increases and overruns to the ventricular area. In **h,** the tumors overrun all right brain mice's cortex, ventricle, and striatum. In **i** and **j**, the tumor shows ventral and dorsal hippocampus. In **k-o,** brain coronal slices anteroposterior stereotaxic coordinates of the sections relative to bregma 0.50 and -7 mm from mice bearing CNS tumor after ZIKV_WT treatment don't show a tumor mass. In **p-t**, brain coronal slices anteroposterior stereotaxic coordinates of the sections relative to bregma 0.50 and -7 mm from mice bearing CNS tumor after oZIKV_2k treatment don't show a tumor. Atlas templates were adapted from Paxinos and Watson (1998). Images zoom of 4x.

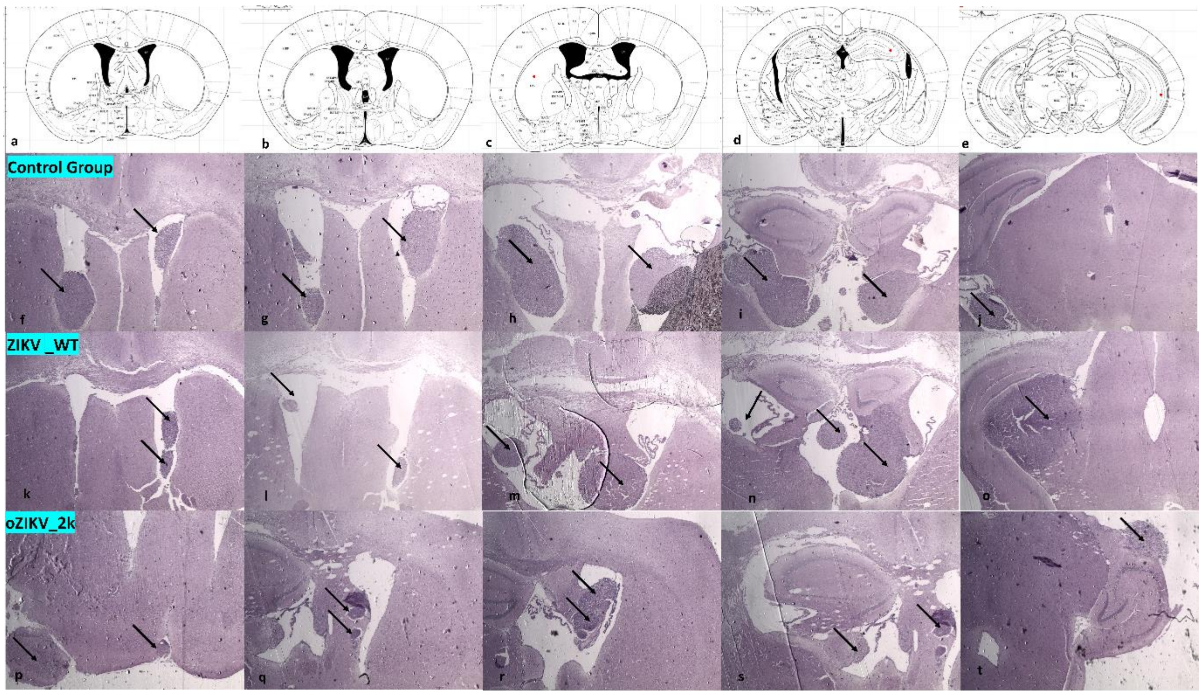

Fig.S6 - Histological images of brain tumor tissue after systemic treatment

In **a-e,** Brain coronal slices anteroposterior stereotaxic coordinates of the sections relative to bregma 0.50 and -7 mm. The **black arrows** indicate tumors in the different brain regions of mice. In **f-h,** the tumor shows inside the ventricular area on both sides. In **i,** the tumor shows the ventral hippocampus area. In **j,** the tumor is localized below the dorsal hippocampus area. In **k-m,** the tumor shows inside the ventricular area on both sides. In **n,** the tumor is localized below the ventral hippocampus. In **o,** the tumor is localized beside the dorsal hippocampus. In **p,** the tumor shows a third ventricle and below the striatum area. In **q,** the tumor is inside the choroid plexus. In **r,** the tumor shows inside the ventricular area. In **s,** the tumor is localized to the ventral hippocampus. In **t,** the tumor is localized above the dorsal hippocampus. Atlas templates were adapted from Paxinos and Watson (1998). Images zoom of 4x.
